# Commensal bacteria antigen-mediated immune response enhances anti-tumor immunity

**DOI:** 10.1007/s00262-025-04275-x

**Published:** 2025-12-24

**Authors:** Jessica Tzu-Chieh Lee, Soo Ngoi, Brian Deng, Megan Hill, Kai He, Yi Yang, Bei Liu

**Affiliations:** 1 Division of Hematology, Department of Internal Medicine, The Ohio State University Comprehensive Cancer Center, Columbus, OH 43210 USA; 2 The Pelotonia Institute for Immuno-Oncology at, The Ohio State University Comprehensive Cancer Center, Columbus, OH 43210 USA; 3 Division of Medical Oncology, The Ohio State University Comprehensive Cancer Center, Columbus, OH 43210 USA; 4 Biocytogen Pharmaceuticals Co, Ltd, Beijing, China

**Keywords:** Commensal, Th17 cell, *Segmented filamentous bacteria*, Tumor immunity, Immunotherapy, Gut–lung axis

## Abstract

Immunotherapy has transformed cancer treatments, but the majority of cancer patients would inevitably develop resistance to immunotherapy. Th17 cells play complex but crucial roles in anti-cancer immune response, although their therapeutic potential remains underutilized. *Segmented filamentous bacteria* (*SFB*) function as prototypical commensal bacteria that can induce intestinal Th17 cells and impact host immune response. In this study, we investigated how *SFB* antigen-mediated immune responses modify the tumor microenvironment and enhance anti-tumor efficacy through a coordinated gut–lung immunological axis. We engineered B16F1 melanoma cells to express either the SFB3340 epitope (B16-3340, an I-A^b^-restricted epitope derived from *SFBNYU_003340* and recognized by 7B8 TCR) or a control vector (B16-MEM) to evaluate *SFB* antigen effects on tumor immunogenicity. We found that expression of the *SFB* epitope in cancer cells decreased the number of lung tumor nodules, and *SFB* colonization further reduced tumor growth in a lung metastasis model. In addition, Th1, Th17, and CD8^+^ Tc1 cells were all increased in the lungs of the B16-3340 tumor-bearing mice compared with B16-MEM control tumor-bearing mice without triggering a compensatory expansion of immunosuppressive Tregs. Interestingly, *SFB* triggers systemic metabolic changes and an increase metabolites from aromatic amino acid degradation pathways, providing biochemical evidence for a functional gut–lung conduit, which integrates innate microbial detection with adaptive tumor-specific immunity. Our research provides evidence to further investigate and develop novel cancer immunotherapies that utilize microbial antigens and microbiome modifications to improve patient outcomes.

**Supplementary Information:**

The online version contains supplementary material available at 10.1007/s00262-025-04275-x.

## Introduction

Cancer immunotherapy has revolutionized cancer treatment by achieving remarkable long-term outcomes for diseases that were once considered incurable [1–6]. The promising potential of immunotherapy faces significant challenges because clinical results show wide variations between different cancer types and within patients who have the same cancer [7–12]. The diverse therapeutic outcomes require researchers to identify multiple influencing factors for treatment effectiveness to expand immunotherapy benefits to more patients.

The immune system requires Th17 cells to maintain proper function at mucosal surfaces [13–15]. These cells function in two opposing ways during cancer development [14–16]. The immune response of Th17 cells promotes tumor growth through angiogenesis and immunosuppression in particular cancer types, but simultaneously strengthens anti-tumor immunity by drawing cytotoxic T cells and activating natural killer cells in different cancer types [14, 17–19]. The progression of cancer depends on Th17 cells in complex ways, but recent studies demonstrate their ability to improve immune checkpoint blockade treatment outcomes [20–22]. The combination of Th17-inducing dendritic cell vaccines with ICB resistance treatment shows promise in ovarian cancer [23], and IL-17A frequencies above a certain threshold predict better responses to anti-PD-1 therapy in melanoma patients [21] and Th17-enhancing approaches transform non-responsive tumors into checkpoint inhibition targets [24]. The different functions of Th17 biology remain unclear since the functions of Th17 cells determined by various factors in the tumor microenvironment (TME) have not been identified, which hinders their use as therapeutic regimens.

The commensal *segmented filamentous bacteria (SFB)* induce intestinal Th17 cells through epithelial attachment and antigen-specific stimulation [25–29]. The Th17 cells generated by *SFB* exhibit unique transcriptional and functional features that distinguish them from Th17 responses triggered by pathogens while maintaining both mucosal defense and immune homeostasis [30–32]. The gut–lung axis communication pathways enable *SFB*-induced Th17 cells to migrate from the gut to peripheral organs, including the lung [31, 33, 34]. The systemic distribution of *SFB*-induced Th17 cells, together with their antigen-specific recognition abilities, makes them suitable candidates for therapeutic applications in anti-tumor immunity. The relationship between microbiome-primed Th17 immunity and cancer immunotherapy through epitope engineering strategies remains unexplored, which creates a significant knowledge gap between microbiome immunology and cancer immunotherapy.

Here, we reveal how *SFB*-mediated Th17 immune responses transform the TME through a gut–lung immunological axis to achieve strong anti-tumor effects. The metastatic niche presentation of *SFB* epitopes activates tumor-infiltrating Th17 cells strongly, which then initiate a complex anti-tumor cytokine cascade through increased IL-17A and IFN-γ production by CD4^+^ T cells and CD8^+^ T cells, and natural killer cells. The epitope-driven immune activation creates a synergistic bond between bacterial commensal immunity and tumor surveillance that links innate microbial recognition to adaptive cancer responses. Comprehensive metabolomics analysis reveals that *SFB*-mediated metabolic reprogramming generates a systemic biochemical environment that enhances anti-tumor immunity through coordinated changes in aromatic amino acid metabolism and microbial-derived compounds across multiple tissue compartments. The integrated research approach shows promise for developing advanced cancer immunotherapies that utilize natural host microbiome immunomodulatory capabilities to enhance current cancer treatment outcomes.

## Materials and methods

### Mice

C57BL/6 J mice were purchased from The Jackson Laboratory (Bar Harbor, ME, USA) and maintained according to the established guidelines. All animal procedures were conducted in accordance with protocols approved by the Institutional Animal Care and Use Committee (IACUC) at the Ohio State University and adhered to the National Institutes of Health Guide for the Care and Use of Laboratory Animals. Mice were routinely monitored for the presence of *SFB* through fecal screening. *SFB*-positive C57BL/6 mice were obtained from Taconic Biosciences (Rensselaer, NY, USA).

### Reagents

Antibodies used for flow cytometry were obtained from BD Biosciences (Mountain View, CA), eBioscience (San Diego, CA), BioLegend (San Diego, CA), TONBO Biosciences (San Diego, CA), and Thermo Fisher Scientific (Waltham, MA) (**Supplemental Table 1**). All other chemicals were obtained from Sigma-Aldrich and Fisher Scientific.

### *SFB* screening by quantitative qPCR

A fecal pellet was crushed in 2 ml of PBS and filtered through a 40 μm cell strainer. The FastPrep-24™ Classic bead beating grinder and lysis system (MP Biomedicals) was used for sample homogenization with 0.1 mm zirconia/silica beads at a speed of 6.5 for 1 min. Bacterial DNA was isolated from the cell lysate supernatant after spinning down at 15,000 rpm for 1 min and purified with the DNA Clean & Concentrator Kit (Zymo Research). Quantitative PCR was performed using SsoAdvanced Universal SYBR Green supermix (Bio-Rad) with published sequences conducted in triplicate (SFB736F, 5’-GACACTGAGGCATGAGAGCAT-3’; SFB844R, 5’-GACGGCACGGATTGTTATTCA-3’; UnitF340, 5’-ACTCCTACGGGAGGCAGCAGT-3’; UnitR514, 5’-ATTACCGCGGCTGCTGGC-3’). The quantification cycle (Cq) value of the qPCR results of *SFB-*specific 16S rRNA was normalized by the Cq value of eubacteria 16S rRNA.

### Isolation of lymphocytes

The spleens underwent mechanical disruption followed by filtration through a 100-μm cell strainer (BD Biosciences, cat#352,360) before erythrocyte lysis with ACK buffer (155 mM NH_4_Cl, 10 mM KCHO_3_, 0.127 mM EDTA). The right ventricle received 10 mL of perfusion solution containing 0.15 mg/ml heparin before the lung tissue collection. The lung tissue underwent processing through 1–2 mm fragment mincing before being placed in 1.3 mM EDTA solution at 37 °C for 30 min with periodic agitation. The tissue fragments underwent centrifugation at 300 × g for 5 min before being mixed with 40 mL of RPMI 1640 digestion buffer containing 5% FBS, 1 mM MgCl₂, 1 mM CaCl₂, and 0.4 mg/mL collagenase D (Roche, cat#11,088,866,001) for one hour at 37 °C with periodic agitation. The enzymatic process stopped when complete RPMI 1640 medium at room temperature was added to the reaction. The enzymatically treated tissue underwent additional mechanical disruption followed by filtration through 100 μm cell strainers (BD Biosciences, cat#352,360). The Percoll gradients at 44%/67% (Cytiva, cat#17,089,101) were used to enrich lymphocytes through density gradient centrifugation. The cells from the interface were collected with care before being washed twice in PBS containing 2% FBS and then analyzed through flow cytometry.

### Flow cytometry

For analysis of intracellular cytokines, single cell suspensions were treat with PMA (50 ng/mL, Sigma-Aldrich, cat#P8139), ionomycin (500 ng/mL, Sigma-Aldrich, cat#I0634), and brefeldin A (BFA, 10 μg/mL, Invitrogen, #00–4506-51) in complete T-cell medium (RPMI 1640 supplemented with 10% FBS, 2 mM L-glutamine, 1% penicillin/streptomycin, and 55 μM β-mercaptoethanol) at 37 °C in a humidified atmosphere with 5% CO₂ for 2 h. The cells were treatment with anti-CD16/CD32 antibody (1:200, Biolegend cat#101,320) at 4 °C for 10 min to block FcγRII/III receptors before surface staining with fluorochrome-conjugated antibodies for 30 min at 4 °C. The cells underwent viability staining with Ghost Dye Fixable Viability Dye before surface staining and then were fixed and permeabilized using the Foxp3/Transcription Factor Staining Buffer Set (eBioscience, Thermo Fisher Scientific) as per the manufacturer’s instructions before antibody staining. Data were collected with the Cytek Aurora spectral flow cytometer and analyzed by FlowJo software V10 (BD Biosciences).

### Cell culture

The B16F1-MEM (vector control) and B16F1-3340 (SFB epitope engineered) murine melanoma cells (B16F1, ATCC CRL-6323, authenticated by morphological assessment and melanin production) were cultured in Dulbecco’s Modified Eagle’s Medium (DMEM, Gibco, cat#D5796-500 ml) supplemented with 10% heat-inactivated fetal bovine serum (Gibco Fetal Bovine Serum, cat#26,140,087), 100 U/mL penicillin/streptomycin (Gibco, cat#15,140–122 100 ml) at 37 °C in a humidified atmosphere with 5% CO2. Stable transfectants were cultured in the presence of 5 μg/ml puromycin (Sigma, cat#P9620-10 mL) for selection, and the drug was removed from the culture medium 24 h before in vivo experiments. For tumor rejection studies, cells were harvested and washed twice with sterile saline (Cellpro, cat#PB500). Mice received 2 × 10^5^ viable cells/100 μL of sterile saline via tail vein injection.

### Metabolon sample preparation and analysis

The serum collection process began after anesthesia application and cardiac puncture required a 22-gauge needle to extract blood from the heart. The blood samples underwent 30 min of ice exposure to allow coagulation before being spun at 13,000 rpm for 20 min at 4 °C to separate serum. The resulting serum samples underwent immediate freezing in liquid nitrogen before being stored at -80 °C until analysis time. The trachea received surgical exposure through the removal of overlying skin and fascia. The insertion of a sterile catheter into the trachea enabled lung lavage with 1 mL of PBS solution. The collection of BALF samples began immediately after the lavage fluid withdrawal. The BALF supernatant was collected after the samples underwent centrifugation at 3,200 rpm for 5 min at 4 °C. The Bradford assay (Bio-Rad, cat#5,000,006) served to measure total protein concentration in BALF samples according to the provided manufacturer’s instructions. Fecal Sample Collection: Fresh fecal samples underwent immediate freezing in liquid nitrogen before storage at -80 °C until further analysis. The researchers conducted metabolomics analysis on seven biological replicate mouse samples (n = 7), which served as experimental subjects. Metabolon Inc. (Morrisville, NC, USA) conducted comprehensive metabolomics profiling on its established global metabolomics platform. The analytical platform combined UHPLC-MS/MS technology with four analytical methods to achieve maximum metabolome coverage through the use of (+ ESI)1, (+ ESI)2, (-ESI), and UHPLC(HILIC)-MS/MS. The analytical run received quality control through process blanks and pooled matrix samples as technical replicates as well as internal standard additions for instrument performance monitoring. The identification of significantly altered metabolites required two statistical filters which started with fold-change criteria of > 2.0 for upregulated metabolites and < 0.5 for downregulated metabolites followed by statistical significance testing (*p*-value < 0.05).

### Statistical analysis

All statistical analyses were performed using GraphPad Prism 9 (GraphPad Software, Inc., California) statistical software packages. All data points represent biological replicates and are presented as mean ± standard error of the mean (SEM). Pairwise comparisons were performed using a 2-way unpaired student’s t-test. A p-value of less than 0.05 was considered statistically significant (*p < 0.05, **p < 0.01, ***p < 0.001, ****p < 0.0001).

## Results

### *SFB* drives Th17 expansion and reprograms immune landscapes in intestinal and lung compartments.

**Fig. 1:**
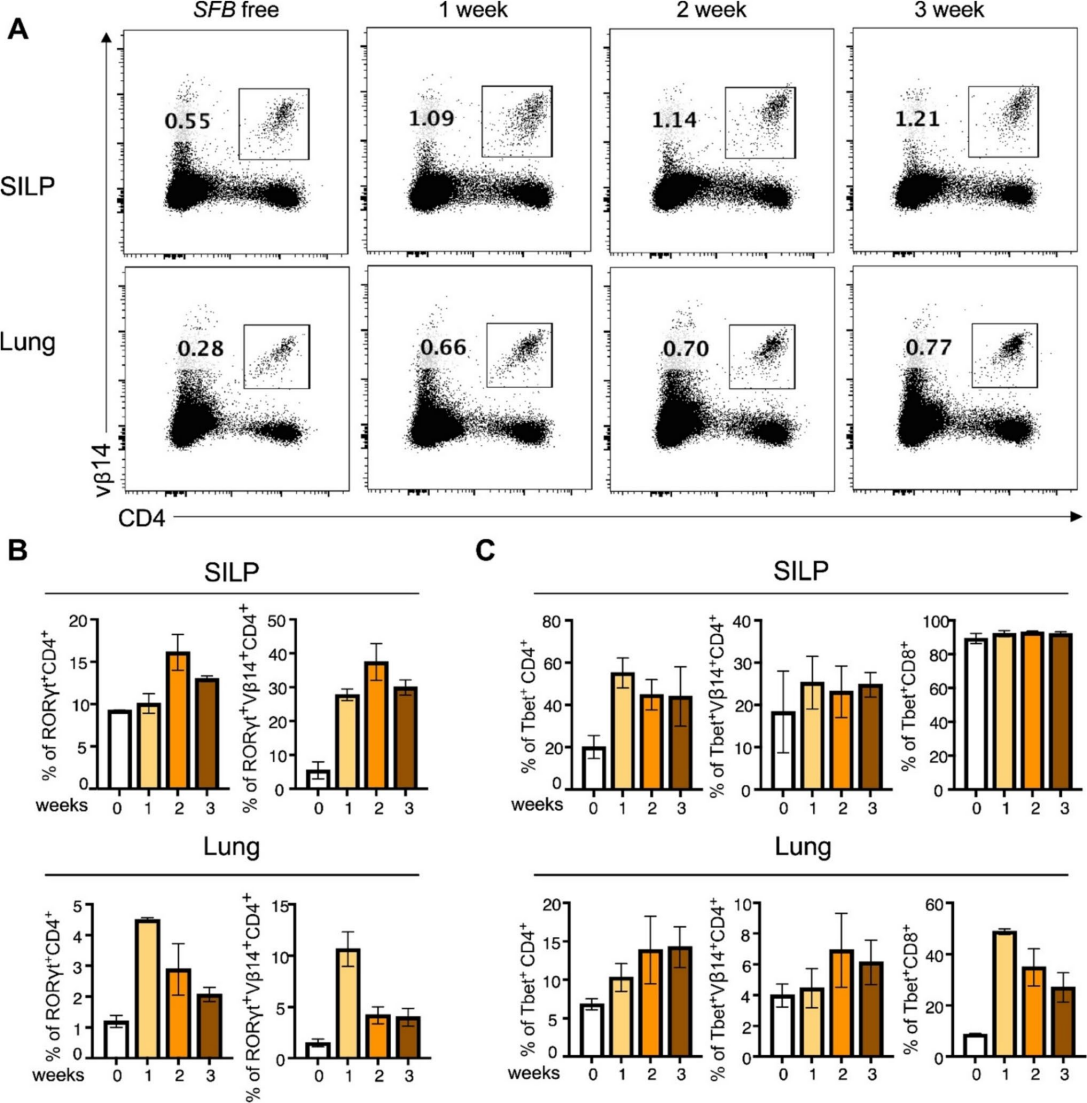
*SFB* drives Th17 expansion and coordinated immune reprogramming in intestinal and lung compartments **A** Representative flow cytometry plots demonstrate the *SFB*-specific Th17 cell (Vβ14^+^CD4^+^) distribution in SILP (top) and lung tissue (bottom) at weeks 0 (n = 2), 1 (n = 2), 2 (n = 3), and 3 (n = 3) following *SFB* colonization. The percentage of Vβ14^+^CD4^+^ T cells within the live lymphocyte gate appears in each plot. **B** The study evaluates RORγt^+^CD4^+^ T-cell differentiation through quantitative methods. The bar graphs present mean population percentages of specific T cells in SILP (top) and lung (bottom) tissues during weeks 1, 2, and 3 following *SFB* colonization. **C** The study evaluates T-bet^+^CD4^+^ and T-bet^+^CD8^+^ T-cell differentiation through time-based analysis. The mean percentage of specific T-cell populations in the SILP (top) and lung (bottom) tissues is shown through bar graphs at weeks 1, 2, and 3 post-*SFB* colonization

To investigate how segmented filamentous bacteria (*SFB*) colonization affects host immune responses through time, we use *SFB*-positive fecal material to inoculate C57BL/6 mice at 1-, 2-, and 3-week intervals and profile the phenotype post-inoculation. We examined immune cell differentiation patterns at two essential sites: the small intestine lamina propria (SILP), where gut microbiota interacts with the host immune system, and lung tissue, which demonstrates established gut–lung communication. The documented restriction of *SFB*-specific Th17 cells to particular T-cell receptor (TCR) repertoires characterized by distinct V-beta chain usage patterns indicates specific antigen recognition of *SFB*-derived antigens presented through MHC class II molecules. The analysis focused on Vβ14^+^CD4^+^ T-cell populations because they serve as markers for *SFB*-responsive immunity. The analysis of *SFB* colonization through oral gavage showed that both SILP and lung tissue developed higher Vβ14^+^CD4^+^ T-cell frequencies than *SFB*-free control groups. The immune priming process became more pronounced through time as the expansion of cells increased during prolonged *SFB* colonization (Fig. 1A). The immune profile showed extensive coordinated changes throughout both tissue compartments when we studied T-cell differentiation under *SFB* colonization conditions. The frequencies of Th17 cells increased substantially in SILP and lung tissues, while this growth occurred in both V*β*14^−^CD4^+^ and Vβ14^+^CD4^+^ T-cell subpopulations (Fig. 1B).

Th17 cells have been shown to gain IFN-γ production capabilities and express T-bet in inflammatory environments where IL-12 signaling dominates, and TGF-β levels decrease. The Th17-to-Th1 plasticity has been shown to enhance antimicrobial immunity and anti-tumor responses; thus, *SFB*-induced Th17 cells may also contribute to broader immune surveillance functions [15, 35]. Our results showed that Th17 cell populations underwent expected phenotypic changes, but additional immune modulation patterns were discovered. T-bet expression levels increased substantially in multiple T-cell subsets, including Vβ14^−^CD4^+^ and Vβ14^+^CD4^+^ populations, as well as CD8^+^ T cells found specifically in lung tissue (Fig. 1C). The discovery indicates that *SFB* colonization activates both Th17 responses and Th1-type immunity priming, which creates an enhanced, versatile immune environment through direct Th1 induction and Th17-to-Th1 transdifferentiation mechanisms [36]. These findings demonstrate that *SFB* colonization serves as a strong immunomodulatory agent, which generates effects that reach beyond gut-specific immune responses. *SFB*, like other commensal bacteria, triggers immune responses by activating specific T cells and performing systemic immune system changes [37].

### Expression of *SFB* epitope in cancer cells reduces metastatic tumor burden and enhances Th17 cell infiltration in a lung metastasis model

**Fig. 2:**
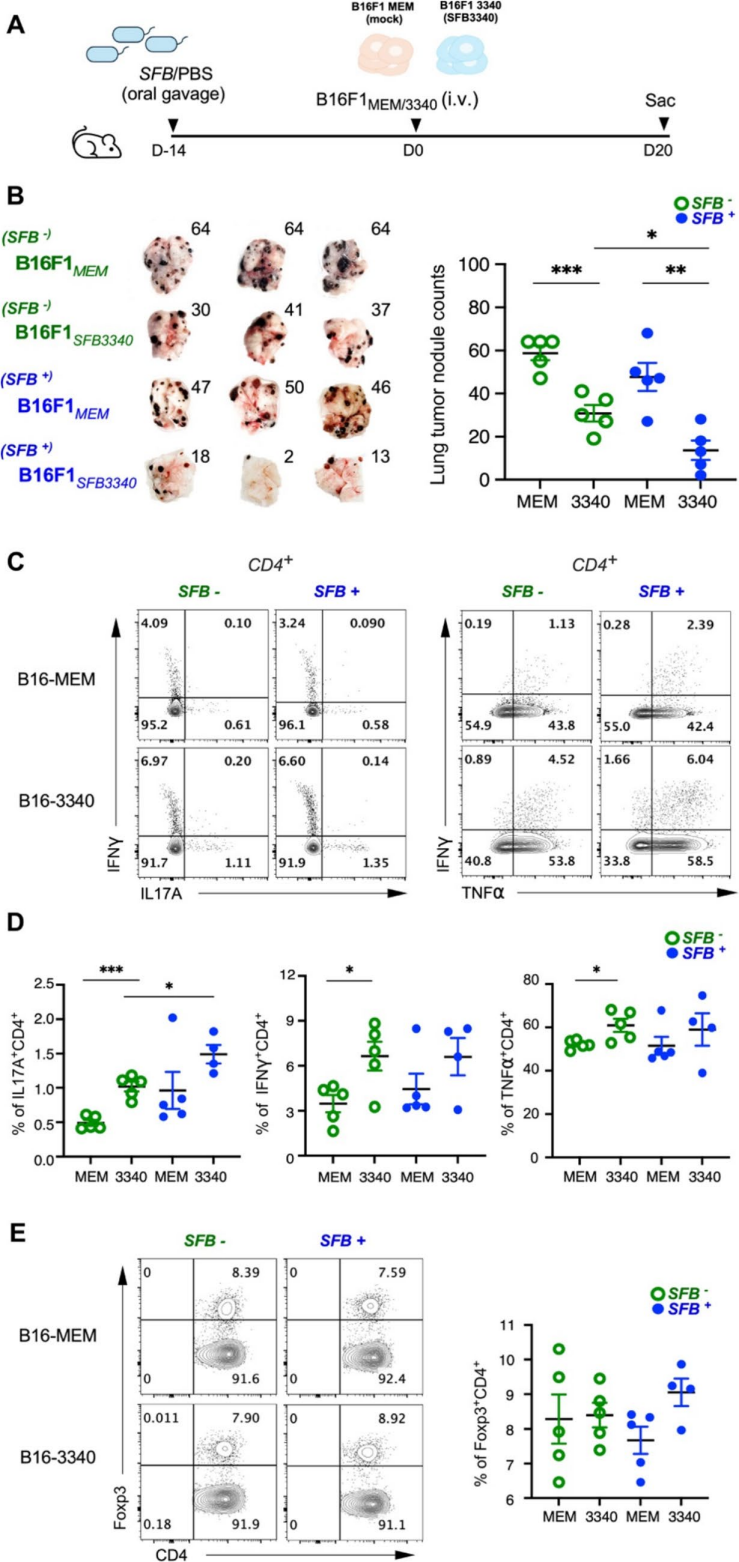
Expression of *SFB* epitope reduces metastatic burden and enhances Th17 infiltration in melanoma. **A** The experimental design follows this schematic workflow. Mice received *SFB* or PBS control through oral gavage before receiving intravenous B16-*SFB*3340 or B16-MEM (control) melanoma cells to create lung metastasis. **B** Left: The lung metastatic nodules in *SFB*-colonized mice exposed to B16F1-*SFB*3340 show a higher tumor burden than control conditions. Right: The quantitative assessment of lung tumor burden is presented as the average number of nodules found in each lung. **C** The flow cytometry results display IL-17A, IFN-γ, and TNF-α expression in CD4^+^ TILs extracted from lung metastases throughout all experimental groups (*SFB*^+^/B16-3340, *SFB*^+^/B16-MEM, *SFB*^−^/B16-3340, *SFB*^−^/B16-MEM). The numbers represent the percentage of cells that fall within each gate. **D** The quantitative evaluation of CD4^+^ T cells producing effector cytokines in lung metastases. **E** Representative flow cytometry plots showing Foxp3 expression in CD4^+^ TILs extracted from lung metastases throughout all experimental groups (left) and quantification of CD4^+^ Foxp3^+^ Tregs (right). n = 4–5 mice per group. Data are shown as mean ± SEM. *p < 0.05, **p < 0.01, **p < 0.001. (2-way unpaired t-test)

Our collaborator previously demonstrated that the TCR of 7B8 TCR was enriched in Th17 cells and identified an *SFB-*derived, I-A^b^ restricted epitope (SFB3340) [26]. To assess the *in vivo* functional impact of *SFB*-driven immune responses, we utilized the B16F1 melanoma cell line, a transplantable tumor model that forms lung tumors following intravenous injection. We engineered these cells to present the SFB3340 epitope (creating the B16-3340 cell line), thereby enabling recognition by *SFB*-primed Th17 cells; a control cell line lacking the epitope (B16-MEM) was generated in parallel. C57BL/6 mice were preconditioned with either *SFB*-positive fecal material or a PBS control via oral gavage for two weeks to establish differential gut colonization. These mice were then intravenously inoculated with either B16-3340 or B16-MEM cells to establish a metastatic lung cancer model, with analysis on day 20 post-tumor inoculation (Fig. 2A). The combination of *SFB* gut colonization and tumor-specific *SFB* epitope expression led to a substantial reduction in pulmonary tumor burden compared to all control groups. Quantification of lung tumor nodules confirmed these strong anti-tumor effects, indicating that SFB3340 epitope recognition and microbiota-primed immunity act synergistically to limit tumor growth (Fig. 2B). Furthermore, adoptive transfer of *SFB*-specific 7B8 T cells into SFB^+^ mice and the combination of SFB epitope expression in tumor cells confirm antigen-driven Th17 recruitment in the lung TME and an anti-tumor response (Fig. S1).

To characterize the immunological mechanism underlying this protection, we analyzed CD4^+^ tumor-infiltrating lymphocytes (TILs). The frequency of IL-17A^+^ CD4^+^ T cells was substantially higher in mice with both *SFB* colonization and SFB3340-expressing tumors (*SFB*^+^ B16-3340) compared to all control groups (Fig. 2C, 2D). Furthermore, the bacterial epitope recognition enhanced broader tumor immunogenicity, inducing robust production of TNF-α and IFN-γ in CD4^+^ T cells alongside the expected Th17 response. Given this pronounced inflammatory boost, we assessed whether it triggered compensatory immunosuppression by quantifying Foxp3^+^ regulatory T cells (Tregs). Notably, Treg frequency remained stable across all groups despite the significant expansion of effector populations (Fig. 2E). These data indicate that the *SFB* antigen activates a potent, multi-functional anti-tumor response, engaging both Th17 and Th1-type immunity, without inducing counter-regulatory Treg expansion. Consequently, the intratumoral immune balance is shifted decisively toward sustained activation.

### *SFB* antigen increases tumor immunogenicity and enhances anti-tumor immune responses

**Fig. 3:**
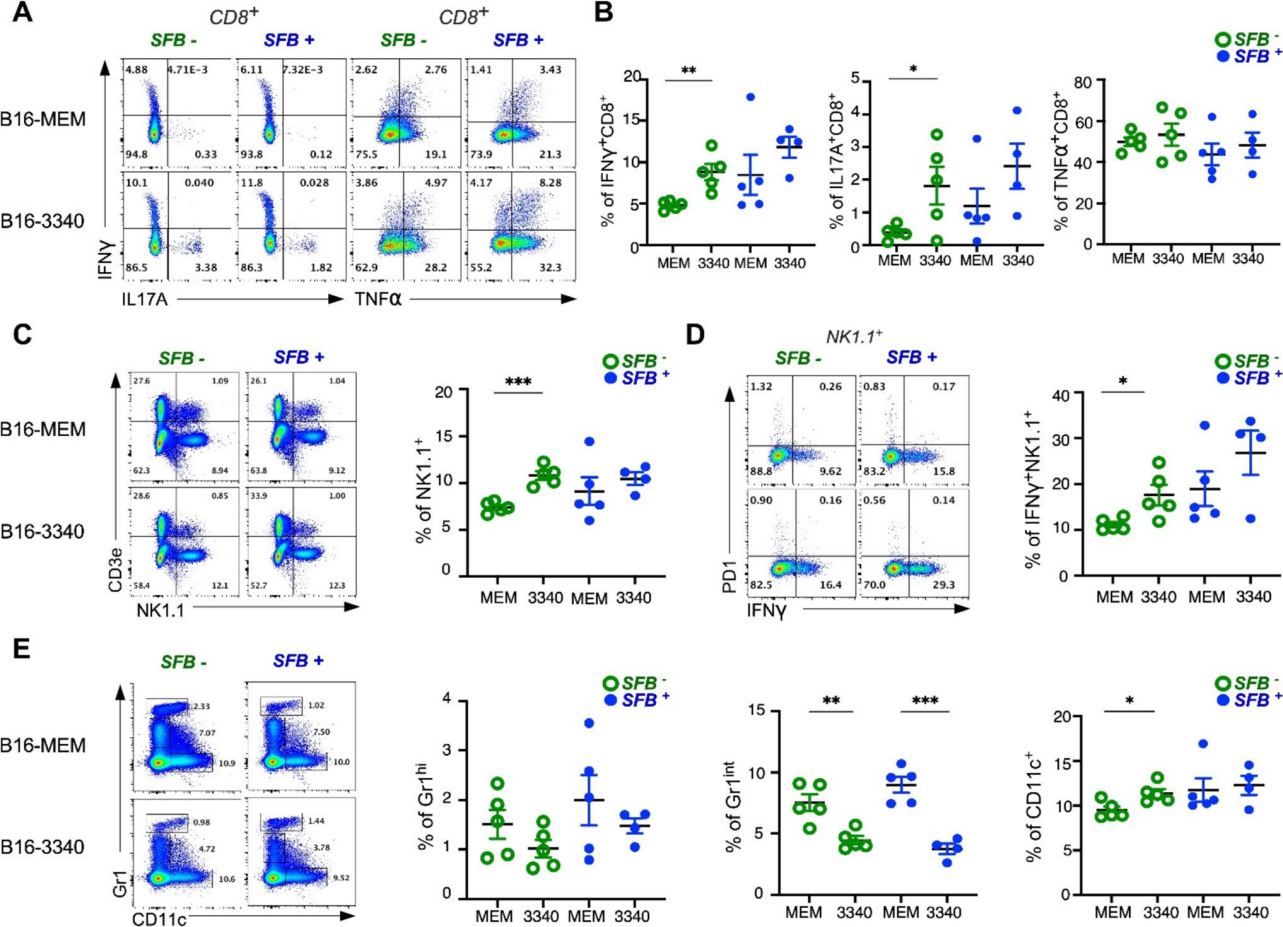
*SFB* antigen increases tumor immunogenicity and enhances anti-tumor immune responses. **A** Representative flow cytometry plots showing IFN-γ, IL17-A, and TNF-α expression in CD8^+^ TILs isolated from lung metastases across all four experimental groups. Numbers indicate the percentage of positive cells in each gate. **B** Quantitative analysis of effector cytokine-producing CD8^+^ T cells within lung metastases. Bar graphs show a mean percentage of IFN-γ^+^, IL-17A^+^, and TNF-α^+^ among CD8^+^ TILs for each experimental group. **C** Representative flow cytometry plots and quantitative analysis of NK cells (CD3^−^NK1.1^+^). **D** Representative flow cytometry plots gated with NK1.1^+^ (left) and quantitative analysis of IFN-γ^+^ NK cells in lung metastases across experimental groups (right). **E** Left: Representative flow cytometry plots showing the frequency of Gr-1^hi^, Gr-1^int^, and CD11c in lung metastases across all four experimental groups. Right: Quantification of Gr-1^hi^, Gr-1^int^, and CD11c^+^ as a percentage of total CD45^+^ cells, with notable trends observed in all four experimental groups. n = 4–5 mice per group. Data were analyzed in a blinded manner and presented as mean ± SEM. *p < 0.05, **p < 0.01, ***p < 0.001. (2-way unpaired t-test)

Th17 cells mediate anti-tumor response through a dual mechanism: recruiting pro-inflammatory immune cells (macrophages, neutrophils, natural killer (NK) cells, and CD8^+^ T cells) while reducing immunosuppressive myeloid-derived suppressor cells (MDSCs) [15, 20]. Flow cytometric analysis of the tumor-infiltrating lymphocytes in our model revealed significant changes in multiple immune cells that collectively enhanced anti-tumor responses in B16-3340 tumor-bearing mice compared to B16-MEM control tumor-bearing mice. The B16-3340 tumor-bearing mice displayed enhanced CD8^+^ T-cell function, marked by a substantial increase in IFN-γ^+^ CD8^+^ and IL17^+ ^CD8^+ ^T cells, indicating robust activation of both  Tc1 and Tc17 effector programs (Fig. 3A, 3B). In contrast, ^+^ TNF-α production by CD8^+^ T cells remained unchanged, suggesting  selective activation of specific cytotoxic pathways rather than a generalized inflammatory state. We also evaluated the innate lymphoid compartment. SFB3340 epitope recognition produced significant changes in NK cells and IFN-γ-producing NK cells within tumor tissues (Fig. 3C, 3D). The activation of NK cells is a critical innate immune response against tumors because they destroy cancer cells and produce IFN-γ to support the adaptive immune response. We next analyzed myeloid cell distribution patterns to assess the TME. Gr-1^int^ MDSCs drive tumor invasion and metastasis through invasive tumor front migration [39, 40]. In B16-3340 tumor-bearing mice, the number of Gr-1^int^ MDSCs was lower than in B16-MEM control tumor-bearing mice, which indicates a crucial shift in the tumor immune environment because decreased MDSC numbers often lead to better immune surveillance and improved tumor responses (Fig. 3E). Furthermore, dendritic cell (DC) populations were elevated in the TME of B16-3340 mice, which facilitates antigen presentation and T-cell activation (Fig. 3E). Together, these changes indicate a reprogrammed TME characterized by heightened cytotoxic activity and reduced myeloid suppression.

### *SFB* antigen bridges gut–tumor immunity

**Fig. 4:**
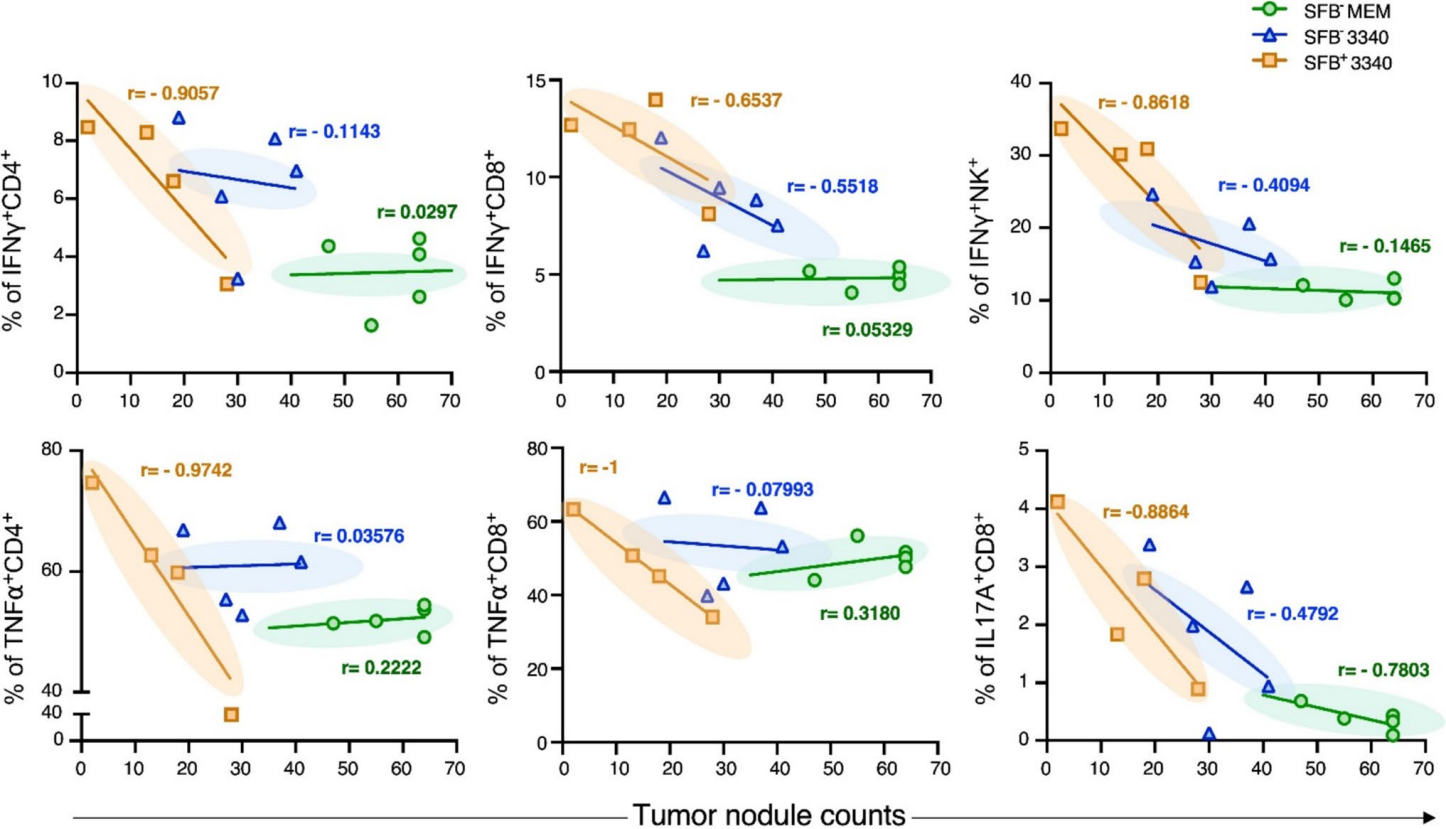
*SFB* antigen bridges gut–tumor immunity. The research investigates the relationship between cytokine-producing tumoricidal immune cell populations and lung tumor burden across various experimental groups. The scatter plots show the correlation between cytokine-producing tumoricidal immune cell populations and lung tumor nodule counts in mice bearing *SFB*^−^B16-MEM (green, n = 5), *SFB*^−^B16-3340 (blue, n = 5), and *SFB*^+^B16-3340 (orange, n = 4) tumor cells. The Pearson correlation coefficients (r) measure linear relationships between variables across each experimental group through values that extend from -1 for a negative perfect correlation to + 1 for a positive perfect correlation. The data points represent individual mice, while trend lines show the correlation patterns within each treatment group

To determine how *SFB* colonization and SFB3340 epitope expression jointly dictate anti-tumor efficacy, we correlated immune cell infiltration with pulmonary tumor burden across all experimental groups. Unlike *SFB*^−^B16-MEM and *SFB*^−^B16-3340, which showed weak or negligible associations, the *SFB*^+^B16-3340 tumor-bearing mice exhibited a consistent and robust inverse correlation across all effector subsets analyzed (Fig. 4). In this synergistic setting, the frequency of IFN-γ^+^ and TNF-α^+^ CD4^+^ T cells in the *SFB*^+^ B16-3340 group showed extremely strong negative correlations with tumor burden (r = -0.9057 and r = -0.9742, respectively), indicating a direct linear relationship between Th1 responses and tumor eradication. Similarly, cytotoxic CD8^+^ T cells producing TNF-α (r = -1.0) and IL-17A (r = -0.8864) were strongly predictive of reduced metastasis, confirming the functional relevance of the Tc1/Tc17 axis.

Innate immunity also contributed, as IFN-γ^+^ NK cell frequency correlated negatively with tumor growth (r = -0.8618). These data demonstrate that the SFB3340 epitope, in the context of gut colonization, establishes a functional coupling between immune infiltration and tumor killing. The uniquely strong correlations in the *SFB*^+^B16-3340 group support the concept that *SFB*-derived antigens bridge commensal immunity with anti-tumor surveillance to drive a coordinated and highly effective immune response.

### *SFB*-mediated multi-tissue metabolic reprogramming enhances anti-tumor immunity

**Fig. 5:**
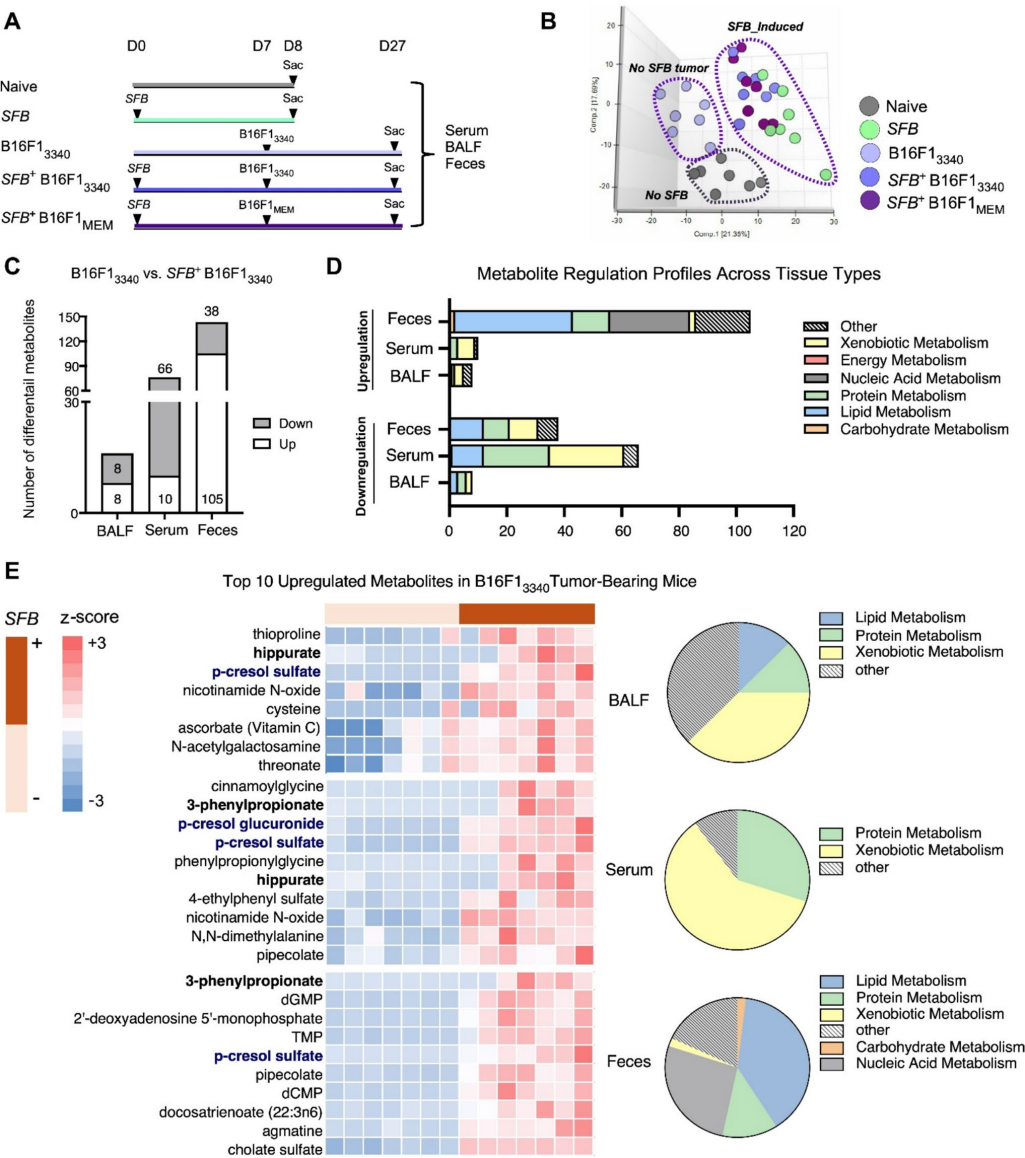
*SFB*-driven systemic metabolic reprogramming enhances anti-tumor immunity.** A** The schema shows the collection process of the serum, BALF, and fecal samples from Naïve, *SFB*^+^, *SFB*^−^B16-3340, *SFB*^+^ B16-3340, and *SFB*^+^ B16-MEM mice (n = 7 mice per group). **B** The PCA score plot demonstrates metabolomics clustering of sample groups based on PC1 versus PC2. **C** Quantitative analysis of differential metabolites between *SFB*^−^ and *SFB*^+^ B16-3340 tumor-bearing mice across serum, BALF, and feces. **D** The analysis of metabolite categories shows the distribution of upregulated and downregulated metabolites by functional class between *SFB*^−^ and *SFB*^+^ B16-3340 tumor-bearing mice across serum, BALF, and feces. **E** The top 10 upregulated metabolites in *SFB*^−^ and *SFB*^+^ B16-3340 tumor-bearing mice are shown in a heat map across all three sample types (serum, BALF, and feces) with individual pie charts illustrating the upregulated metabolic pathway composition for each biological compartment

We have shown that *SFB* colonization further strengthens anti-tumor immune responses. We seek to establish whether *SFB* impacts the functionality of the immune system through alterations of metabolic homeostasis during *in vivo* experiments. A complete analysis of global metabolites occurred across different biological spaces. Untargeted metabolomics analysis of *SFB*-negative versus *SFB*-positive mice enabled the study of biochemical processes in microbiota-immune interactions. Biological samples came from seven mice in each of the five experimental groups (n = 7/group), which provided enough biological replicates for complete metabolomics profiling between naïve controls and *SFB*-colonized mice as well as between *SFB*-negative B16-3340 tumor-bearing mice (*SFB*^−^B16-3340) and *SFB*-colonized B16-3340 tumor-bearing mice (*SFB*^+^B16-3340) and *SFB-*colonized B16-MEM control tumor-bearing mice (*SFB*^+^B16-MEM), thus enabling complete evaluation of metabolic changes within different colonization and tumor settings (Fig. 5A). A principal component analysis (PCA) was applied to the complete metabolomics dataset to visualize global metabolic patterns and identify differences between experimental groups. The PCA analysis separated *SFB*-negative from *SFB*-positive individuals through distinct clustering patterns that confirmed *SFB* colonization triggers substantial host metabolic changes, which extend throughout the entire metabolome (Fig. 5B). The comparison of *SFB*^−^B16-3340 versus *SFB*^+^B16-3340 groups showed substantial metabolic changes, which occurred in bronchoalveolar lavage fluid (BALF), serum, and fecal samples (Figs. 5C, 5D). The multi-tissue metabolomics signature demonstrates that *SFB* colonization creates systemic metabolic changes that affect gut metabolism as well as circulating systems and respiratory tract biochemistry, which could be associated with host immune modulation and anti-tumor responses.

To identify systemically altered metabolites, we analyzed the top 10 most significantly increased metabolites across all tissue compartments in SFB-colonized, B16F1-3340 tumor-bearing mice versus non-colonized controls. Heatmap analysis with z-score standardization was used to visualize metabolite intensities for direct comparison of abundance patterns between organs and tissues, with metabolites ordered by their fold-change magnitude from highest to lowest. We observed increased concentrations of metabolites associated with the aromatic amino acid degradation pathway, including hippurate and its upstream precursor, 3-phenylpropionate (Fig. 5E, highlighted in black), suggesting enhanced microbial degradation of phenylalanine and tyrosine. The metabolic breakdown of p-cresol into its conjugated forms, p-cresol sulfate, and p-cresol glucuronide, was elevated significantly (Fig. 5E, highlighted in blue). The concurrent elevation of these gut-derived metabolites in serum and lung (BALF) provides direct biochemical evidence of a functional gut–lung conduit, confirming that microbial signals are physically traversing to the metastatic niche. Notably, elevated hippuric is linked to improved response to PD-1 blockade in non-small cell lung cancer [41], supporting the translational relevance of this conserved metabolic signature. While causal roles require further study, the robust systemic presence of these metabolites defines a specific immunometabolic state and highlights their potential as non-invasive biomarkers for monitoring the gut–lung immunological axis.

## Discussion

The immunological function of Th17 cells within tumors continues to be debated since both pro-tumorigenic and anti-tumorigenic actions have been observed [17, 42]. Research has shown that Th17 cells support tumor growth through mechanisms that include tumor cell migration and metastasis, as well as angiogenesis and regulatory T-cell recruitment, and through the release of immunosuppressive cytokines IL-10 and TGF-β [14, 43–45]. These immune activities help create a tumor-supportive environment and lead to worse outcomes in various cancer types. Numerous scientific investigations have also shown that Th17 cells possess the capability to create anti-tumor immunity. The cytokines released by Th17 cells activate cytotoxic immune cells, including CD8^+^ T cells and natural killer (NK) cells, and bring anti-tumor macrophages into the fight [46–48]. Furthermore, Th17 cells demonstrate plasticity by converting into Th1-like cells, which increases their tumor-killing properties by generating IFN-γ and additional inflammatory agents [14].

To translate this mechanism into a therapeutic strategy, we leveraged the *SFB*–host interaction as a model system. We redirected gut-primed Th17 cells against metastatic lung cancer by engineering B16F1 melanoma cells to express the SFB3340 epitope. We demonstrate that a commensal-derived antigen can function as a powerful immunological bridge, directly coupling pre-existing mucosal immunity to adaptive anti-tumor surveillance.

Our study establishes a mechanistic proof-of-principle for the “Gut–Lung Immunological Axis” in cancer control. We demonstrate that the combination of *SFB* gut colonization with tumor-specific epitope presentation led to a substantial reduction in metastatic burden, driven by a coordinated expansion of Th17, Th1, and cytotoxic CD8^+^ T cells. Crucially, our analyses revealed that this inflammatory boost occurred without a compensatory expansion of Foxp3^+^ Tregs. Consequently, the treatment significantly improved the effector-to-regulator ratio within the TME, a critical determinant of therapeutic success. Furthermore, using adoptive transfer of *SFB*-specific 7B8 TCR-transgenic T cells, we provided direct evidence that this effect is antigen-specific. Naive gut-specific T cells were capable of trafficking to the lung and differentiating into IL-17A-producing effectors only when the appropriate microbial signals and tumor antigens were present. Regarding the mechanism of antigen presentation, given that B16F1 cells lack constitutive MHC class II expression, we propose that the SFB3340 epitope is processed via indirect cross-presentation by tumor-infiltrating dendritic cells (DCs), which we found to be functionally licensed in the responder group.

Our metabolomics data provide a critical translational link for these findings. The gut metabolite hippurate is elevated in *SFB*-presenting guts, bronchoalveolar lavage fluid (BALF), and serum. The presence of these gut-derived metabolites in the lung provides biochemical evidence of a functional gut–lung conduit, confirming that microbial signals physically traverse to the metastatic niche. Previous research demonstrated that a particular set of plasma metabolites, including hippuric acid (a product derived from microbiota), could predict therapeutic response to PD-1 blockade immunotherapy (AUC = 0.91). The combination of T-cell markers, including mitochondrial activation with CD8⁺PD-1^hi^ and CD4⁺ T-cell frequencies, led to an even more accurate prediction (AUC = 0.96) [41]. It was also previous reported that hippuric acid levels have been linked to better anti-tumor responses and enhanced clinical outcomes in patients receiving PD-1 blockade therapy and these metabolites from microbiota could activate host immune cells [49].

While this study establishes a critical role for *SFB*-induced, Th17-mediated immunity in controlling metastatic lung cancer via a gut–lung axis, several key limitations point to future directions. First, as *SFB* is a mouse-specific commensal, translating this strategy requires identifying homologous Th17-inducing epitopes from human commensals such as *Bifidobacterium* or *Bacteroides* species. Second, although we identified a strong correlation between the metabolite hippurate and tumor regression, its functional role in T-cell licensing requires definitive validation through perturbation studies (e.g., dietary supplementation or bacterial genetic manipulation). Despite these limitations, our work demonstrates that microbiota can systemically reshape metabolism and the immune microenvironment to integrate innate microbial sensing with adaptive anti-tumor immunity. These findings provide a foundational rationale for developing novel cancer immunotherapies that engineer commensal-based antigens to target difficult-to-treat lung metastases.

## Supplementary Information

Below is the link to the electronic supplementary material.

Supplementary file1 (PDF 664 KB)

Supplementary file2 (PDF 23 KB)

## Data Availability

The datasets generated during the current study are available from the corresponding author upon reasonable request.
