# Supplementary material for: Commensal bacteria antigen-mediated immune response enhances anti-tumor immunity
Source: Cancer Immunol Immunother. 2025 Dec 24;75(1):28. doi: 10.1007/s00262-025-04275-x (PMC12738396; doi:10.1007/s00262-025-04275-x)
Supplement: Supplementary file 1 — Supplementary file1 (PDF 664 KB) [file 262_2025_4275_MOESM1_ESM.pdf]

Supplemental Figure legend

Figure S1. Adoptive transfer of *SFB*-specific 7B8 T cells confirms antigen-driven Th17 recruitment and anti-tumor response. (A) Schematic illustration of experimental design. *SFB* negative C57BL/6 mice were colonized with *SFB* via oral gavage (day -14), receiving an intravenous transfer of naive $CD45.1^+$ 7B8 TCR-transgenic $CD4^+$ T cells (day -7), and were challenged with B16-MEM (control) or B16-*SFB3340* (epitope-expressing) tumor cells (day 0). **(B)** Lung tumor burden. Representative images (left) and nodule quantification (right) from the indicated groups. **(C)** Donor $CD4^+$ 7B8 T cells recruitment. Representative flow cytometry plots (left) and quantification of donor-derived $CD45.1^+$ 7B8 $CD4^+$ T cells within the lung tumor microenvironment (right). Data are presented as mean ± SEM. $^{**}p < 0.01$. (2-way unpaired t-test).

Figure S1

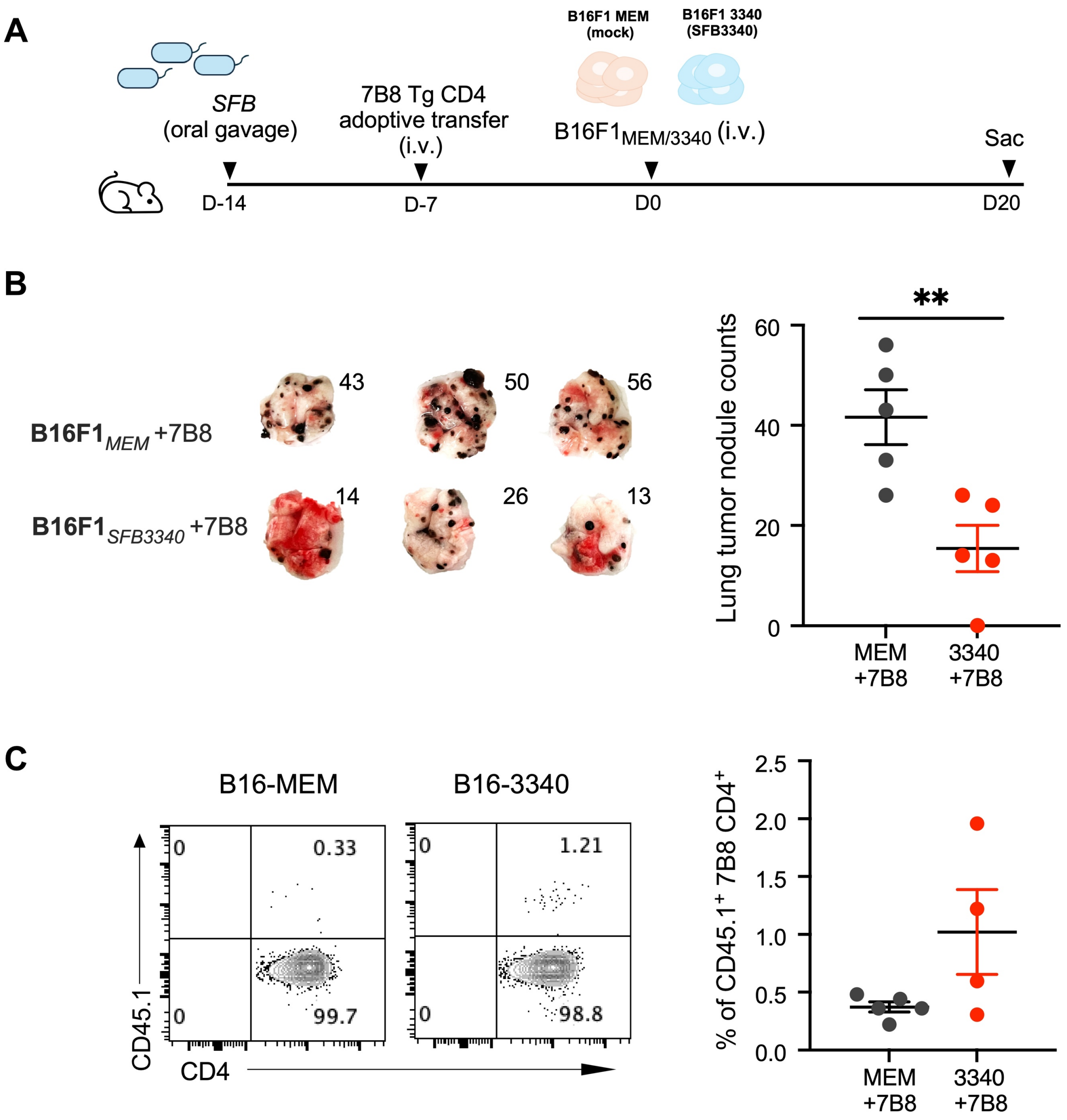
A
B16F1 MEM
(mock)
B16F1 3340
(SFB3340)
SFB
(oral gavage)
7B8 Tg CD4
adoptive transfer
(i.v.)
B16F1MEM/3340 (i.v.)
Sac
D-14
D-7
D0
D20
B
B16F1MEM +7B8
B16F1SFB3340 +7B8
43
50
56
14
26
13
**
Lung tumor nodule counts
60
40
20
0
MEM
+7B8
3340
+7B8
C
B16-MEM
B16-3340
0
0.33
0
99.7
0
1.21
0
98.8
CD45.1
CD4
% of CD45.1+ 7B8 CD4+
2.5
2.0
1.5
1.0
0.5
0.0
MEM
+7B8
3340
+7B8
