# Supplementary material for: Commensal bacteria antigen-mediated immune response enhances anti-tumor immunity
Source: Cancer Immunol Immunother. 2025 Dec 24;75(1):28. doi: 10.1007/s00262-025-04275-x (PMC12738396; doi:10.1007/s00262-025-04275-x)
Supplement: Supplementary file 2 — Supplementary file2 (PDF 23 KB) [file 262_2025_4275_MOESM2_ESM.pdf]

Supplemental Table 1 List of Reagents

Panel # 1 (SFB colonization phenotype)			
**Antibody for flow cytometry**	**Clone**	**Vendor**	**Catalog number**
BV605 anti-mouse CD4 (1:400)	RM4-5	Biolegend	100548
V450 anti-mouse CD8 (1:400)	53-6.7	Tonbo	75-0081-U100
AF700 anti-mouse CD45 (1:400)	30-F11	BD Biosciences	565478
APC/cy7 anti-mouse IL-2 (1:200)	JES6-5H4	BD Biosciences	560547
FITC anti-Vβ14 (1:400)	14-2	BD Biosciences	553258
PE anti-mouse Rorγt (1:400)	B20	Thermo	12-6981-80
eF660 anti-mouse T-bet (1:400)	4B10	Thermo	50-5825-82
**Panel # 2 (lung metastasis phenotype)**			
**Antibody for flow cytometry**	**Clone**	**Vendor**	**Catalog number**
BUV395 anti-mouse CD4 (1:400)	GK1.5	BD Biosciences	563790
BUV496 anti-mouse CD8 (1:400)	53-6.7	BD Biosciences	750024
BUV805 anti-mouse CD11C (1:400)	N418	BD Biosciences	749038
BV421 anti-mouse TIM3 (1:400)	RM13-23	Biolegend	119723
V450 anti-mouse Gr-1 (1:200)	RB6-8C5	Tonbo	75-5931-U100
BV605 anti-mouse NK1.1 (1:200)	PK136	BD Biosciences	563220
BV711 anti-mouse CD45.1 (1:200)	A20	Biolegend	110739
BV785 anti-mouse TNF-α (1:400)	NP6-XP22	Biolegend	506341
FITC anti-mouse CD3e (1:200)	145-2C11	eBioscience	11-0031-85
PerCP5.5 anti-mouse IL-17A (1:400)	TC11-18H10.1	Biolegend	506920
PE anti-mouse PD1 (1:400)	29F.1A12	Biolegend	135206
PE/cy7 anti-mouse Foxp3 (1:600)	3G3	Tonbo	605773U100
APC anti-mouse IFN-γ (1:200)	XMG1.2	eBioscience	17-7311-82
